# Complete annotated genome sequence of *Microbacterium paraoxydans* phage Evcara, a cluster GI podovirus isolated from compost.

**DOI:** 10.17912/micropub.biology.001398

**Published:** 2024-12-08

**Authors:** Hannah B. Bonogafsky, Kelly M. Freer, Taylor B. Sanderson, Kira A. Yost, Ayden J. Pyle, Ginavieve Rowley, Maria D. Gainey

**Affiliations:** 1 Department of Chemistry and Physics, Western Carolina University, Cullowhee, North Carolina, US

## Abstract

Bacteriophage Evcara is a podovirus isolated on *Microbacterium paraoxydans* NRRL B-24275. Its genome is 16,285 bp in length and contains 22 predicted protein-coding genes. Evcara, has been assigned to cluster GI with *Microbacterium* *foliorum* phages PineapplePizza and Curie that share 10 homologues with the well-characterized *Bacillus subtilis* phage phi29.

**Figure 1. Evcara plaque morphology and cluster GI genomic organization:**
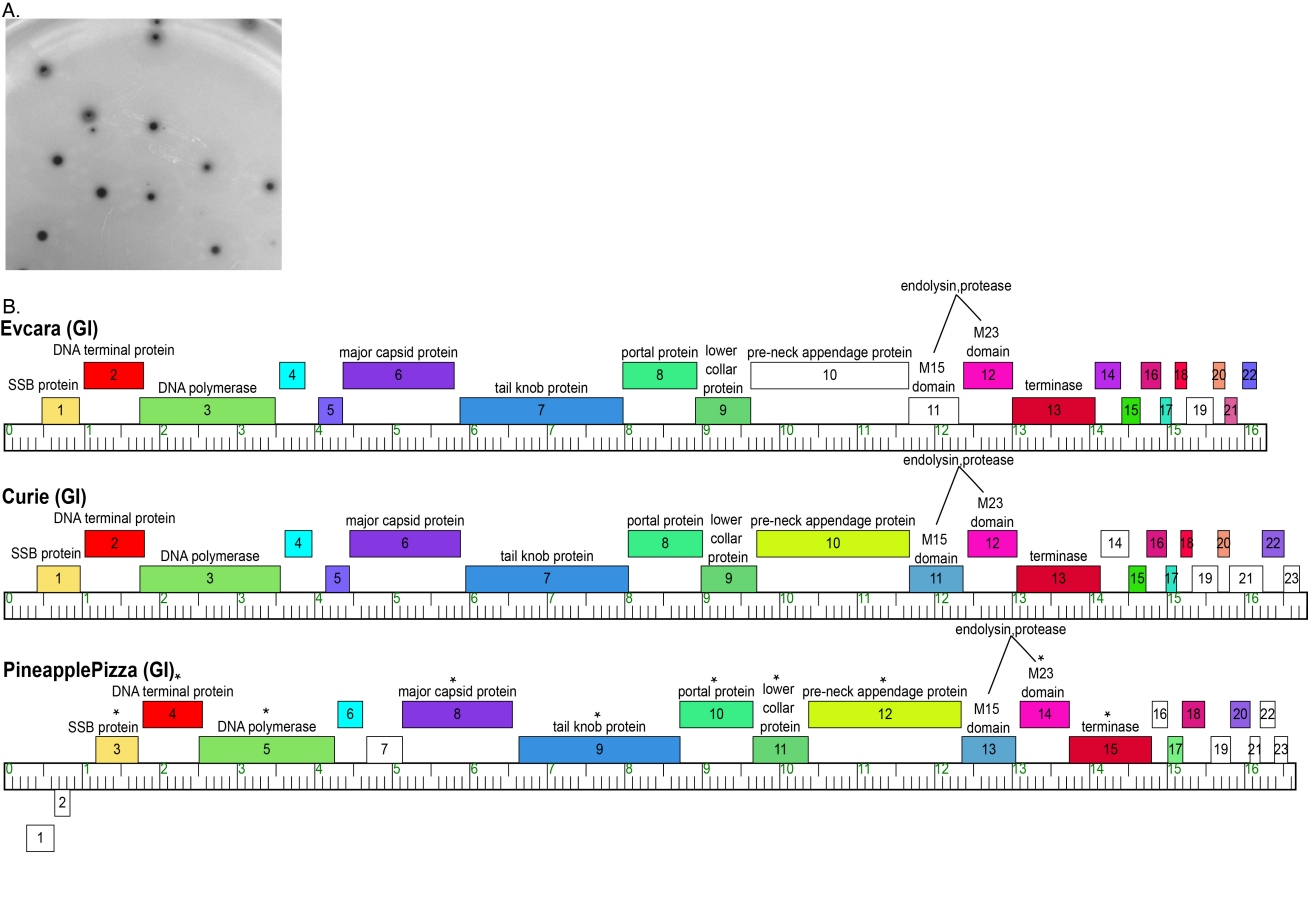
(A) Evcara forms plaques with clear centers and two halos that vary in size (1mm to 6mm in diameter) and halo appearance when plated on *M. paraoxydans* PYCa top agar overlays incubated for 48hr at 30°C. (B) A representation of the genomes of all three cluster GI bacteriophages was generated using Phamerator. Genes are represented with numbered boxes, and assigned functions are listed. Boxes above and below the ruler represent genes that are transcribed rightwards and leftwards, respectively. PineapplePizza genes with phi29 homologues are denoted with a “*.” Genes represented as white boxes are genes with no homologues in the Actinobacteriophage database. &nbsp;

## Description

To date, the Actinobacteriophage database (phagesDB.org), hosts genome sequences for 690 *Microbacterium* phages, isolated using 16 species of *Microbacterium* (Russell & Hatfull, *20* 17 *)* . These phages exhibit considerable genetic diversity, are predominantly lytic, and have been classified into 23 genetic clusters (Jacobs-Sera et al., 2020; Pope et al., 2017) . Here we describe Evcara, a phage isolated using *Microbacterium paraoxydans* NRRL B-24275, one of only three cluster GI bacteriophages isolated, to date.

In the fall of 2023, a sample from the Cullowhee Community Garden compost pile in Cullowhee, North Carolina, USA (GPS 35.317778 N, 83.180833 W) was washed with peptone-yeast calcium (PYCa) media. The wash was filtered (0.22µm) and then enriched by inoculating the filtrate with *M. paraoxydans* . After incubation with shaking (48hrs at 30°C), the culture was filtered and the filtrate spotted onto PYCa plates containing top agar overlays supplemented with *M. paraoxydans* . After incubation at 30°C for 48h, a clearance with a turbid center was observed, indicative of bacteriophage infection. Bacteriophage Evcara was then plated to obtain individual plaques and purified via 5 additional rounds of plating, after which a lysate was prepared (Zorawik et al., 2024) . Evcara plaques vary in size (1mm to 6mm in diameter) and have clear centers encircled by two halos of varying turbidity ( Fig. 1A ).

Genomic DNA was isolated from the lysate using a Promega Wizard DNA cleanup kit and prepared for sequencing using an NEB Ultra II library kit. A total of 561,542, 150bp single-end reads (5,008x genome coverage) were generated using an Illumina MiSeq instrument (v3 reagents) at the Pittsburgh Bacteriophage Institute. Raw reads were assembled into a single contig using Newbler v2.9, and checked for accuracy using Consed v.29 (Gordon & Green, 2013; Margulies et al., 2005; Russell, 2018). Default parameters were used for all software unless otherwise specified.

The Evcara genome is 16,285 bp in length (54.0% GC) with 121bp inverted repeats at the genome ends that are likely covalently bound by terminal proteins as described for phi29 (Yehle, 1978) . Utilizing DNA Master v2705 (http://cobamide2.bio.pitt.edu/), embedded with Genemark v2.5 p and Glimmer v3.02, an automated annotation of the Evcara Genome was generated (Besemer & Borodovsky, 2005; Delcher et al., 2007) . Start sites were manually reviewed using Starterator v1.2 (http://phages.wustl.edu/starterator/), Phamerator v567 (Cresawn et al., 2011) using the Actino_draft v578, and PECAAN v.20240320 (https://discover.kbrinsgd.org/evidence/summary). BLASTp (Altschul et al., 1990) searches against the NCBI non-redundant and Actinobacteriophage databases, and HHpred (Söding et al., 2005) searches against the PDB_mmCIF70_8_Mar, Pfam-A_v37, UniProt-SwissProt-viral70_3, and NCBI_Conserved_Domains(CD)_v3.19 databases were used to assign putative functions. No tRNA genes were identified using Aragorn v1.2.41 (Laslett & Canback, 2004) or tRNAscanSE v.2.0 (Lowe & Chan, 2016) .

Whole-genome alignment using NCBI BLASTn (Altschul et al., 1990) showed no significant nucleotide similarity with any other sequences in NCBI's core nucleotide blast database, with the exception of a 233bp region (81% identity) to *Microbacterium* phage Curie's genome. However, genome annotation revealed extensive shared gene content with *Microbacterium foliorum* podoviruses Curie and PineapplePizza, surpassing the clustering threshold of 35% gene content similarity allowing Evcara to be assigned to actinobacteriophage cluster GI (Pope et al., 2017) . Despite their lack of nucleotide similarity, the 3 cluster GI bacteriophages have a highly conserved genome architecture consisting of 22 to 23 predicted protein coding genes all transcribed in the same direction, with the exception of PineapplePizza's first two genes which are transcribed in the opposite direction ( Fig. 1B ). Evcara has 22 genes, 11 of which were assigned a function. These functions are conserved for all members of cluster GI, 10 of which were previously reported to be homologous to the well-studied podovirus *Bacillus* phage phi29 (Horton et al., 2024; LaBianca et al., 2023; Xu et al., 2019) .

**Nucleotide sequence accession numbers**

GenBank Accession No.: PQ114753

Sequence Read Archive (SRA) No.: SRX25734226
